# Environmental NO_2_ and CO Exposure: Ignored Factors Associated with Uremic Pruritus in Patients Undergoing Hemodialysis

**DOI:** 10.1038/srep31168

**Published:** 2016-08-10

**Authors:** Wen-Hung Huang, Jui-Hsiang Lin, Cheng-Hao Weng, Ching-Wei Hsu, Tzung-Hai Yen

**Affiliations:** 1 Department of Nephrology and Division of Clinical Toxicology, Chang Gung Memorial Hospital, Linkou Medical Center, Taiwan, ROC; 2 Chang Gung University College of Medicine, Taoyuan, Taiwan, ROC; 3 Division of Nephrology, Department of Internal Medicine, Tao-Yuan General Hospital, Ministry of Health and Welfare, Taoyuan, Taiwan, ROC; 4 Graduate Institute of Clinical Medicine, Taipei Medical University, Taipei, Taiwan, ROC

## Abstract

Uremic pruritus (UP), also known as chronic kidney disease–associated pruritus, is a common and disabling symptom in patients undergoing maintenance hemodialysis (MHD). The pathogenesis of UP is multifactorial and poorly understood. Outdoor air pollution has well-known effects on the health of patients with allergic diseases through an inflammatory process. Air pollution–induced inflammation could occur in the skin and aggravate skin symptoms such as pruritus or impair epidermal barrier function. To assess the role of air pollutants, and other clinical variables on uremic pruritus (UP) in HD patients, we recruited 866 patients on maintenance HD. We analyzed the following variables for association with UP: average previous 12-month and 24-month background concentrations for nitrogen dioxide (NO_2_) and carbon monoxide (CO), and suspended particulate matter of <2.5 μm (PM_2.5_). In a multivariate logistic regression, hemodialysis duration, serum ferritin levels, low-density lipoprotein levels, and environmental NO_2_/CO levels were positively associated with UP, and serum albumin levels were negatively associated with UP. This cross-sectional study showed that air pollutants such as NO_2_ and CO might be associated with UP in patients with MHD.

Uremic pruritus (UP), also known as chronic kidney disease–associated pruritus, is a common and disabling symptom in patients undergoing hemodialysis. About 42–90% of patients on dialysis experience pruritus^1^,^2^,^3^. This symptom lasts for months to years in most cases and is resistant to conventional treatments^4^. According to Mettang^5^, as patients with end-stage renal disease also have many other chronic diseases, definitive diagnosis of UP may be challenging. Until now, the pathogenesis of UP was considered multifactorial and still poorly understood^6^,^7^,^8^,^9^. At least 4 main hypotheses have been described, including dermatological abnormalities, an immune-system derangement that results in a proinflammatory state, an imbalance of the endogenous opioidergic system, and a neuropathic mechanism^10^.

Outdoor air pollution has well-known effects on the health of patients with allergic diseases. Exposure to air pollutants is related to neutrophilic inflammation and an increase in cytokine and IgE levels^11^,^12^,^13^, which aggravate dermatitis^14^. In urban areas, air particulates that are enriched with metals have also been believed to contribute to inflammatory responses^15^. Our previous study^16^ showed that residential area was a risk factor for 2-year mortality in patients undergoing hemodialysis, and mean concentration levels of air pollutants (NO_2_, CO and PM_2.5_) were significantly high in basin terrain. We also determined an association among air pollutants and mortality, inflammation, vascular disease, and infection in patients on dialysis^17^,^18^,^19^,^20^. Air pollution–induced inflammation could occur in the skin and aggravate skin symptoms such as pruritus or impair the epidermal barrier function^21^,^22^,^23^. However, to our knowledge, no in-depth study on the correlation between air pollutants and UP in maintenance hemodialysis (MHD) patients has been reported. The aim of this cross-sectional study was to assess the role of residential area, air pollutants, age, male sex, body mass index (BMI), smoking status, diabetes mellitus (DM), hypertension, previous cardiovascular diseases (CVDs), hepatitis B virus (HBV) infection, hepatitis C virus (HCV) infection, HD duration, blood access as arterial-venous(AV) fistula, undergoing hemodialfiltration (HDF), clearance of urea (Kt/V_urea_ Daugirdes), normalized protein catabolism rate (nPCR), non-anuria status, hemoglobin levels, serum albumin levels, serum creatinine levels, corrected calcium levels, inorganic phosphate levels, log-transformed (log) ferritin levels, log intact parathyroid hormone levels (log iPTH), log high-sensitivity C-reactive protein (log hsCRP) levels, low-density lipoprotein (LDL) levels and triglyceride levels on UP in hemodialysis (HD) patients.

## Results

Finally, 866 MHD patients (440 men and 426 women) with a mean MHD duration of 6.96 ± 5.35 y were included in the study. Table 1 lists patient characteristics including age, gender, and BMI, along with the biological, hematological, and HD data for all patients and patients with or without UP. Among the patients, 50.8% were male, 21.8% had UP, 22.2% had a history of DM, 4.7% had CVDs, 2.9% had lupus, 17.3% were habitual users of tobacco, 79.6% had an AV fistula that was utilized, 11.3% had hepatitis B virus infection, and 19.4% had HCV infection.

### Comparison of Clinical Variables between Patients with and without UP

Comparing patients with and without UP, a higher proportion of patients with UP lived in Taipei Basin (65.1% vs 6.6%, respectively; *P* < 0.001), had an HCV infection (24.9% vs 17.9%, respectively; *P* = 0.037), and had undergone HDF (27.5% vs 19.9%, respectively; *P* = 0.028), while a lower proportion had DM (13.2% vs 24.7%, respectively; *P* = 0.001) and a non-anuria condition (11.6% vs 23%; *P* < 0.001). Furthermore, patients with UP had longer HD durations (9.41 ± 5.43 vs 6.27 ± 5.13 y, respectively; *P* < .001), were older (57.93 ± 12.43 vs 55.70 ± 13.86 y, respectively; *P* = 0.047), had higher Kt/V Daugirdes (1.89 ± 0.34 vs 1.77 ± 0.32, respectively; *P* < 0.001), had higher nPCR (1.22 ± 0.27 vs 1.17 ± 0.26 g/kg/day, respectively; *P* = 0.03), had higher iPTH levels (187.2 vs 121.3 pg/mL, respectively; *P* = 0.003), had higher cholesterol levels (176.93 ± 39.24 vs 169.73 ± 37.08 mg/dL, respectively; *P* = 0.02), had higher LDL levels (100.36 ± 31.05 vs 93.26 ± 30.30 mg/dL, respectively; *P* = 0.005), and had lower serum albumin levels (4.01 ± 0.33 vs 4.07 ± 0.34 g/dL, respectively; *P* = 0.019) than patients without UP (Table 1).

### Associations among UP, Clinical Variables, and Residential Area

Univariate logistic regression identified several clinical variables that were significantly associated with UP (Table 2), such as BMI, DM, HCV infection, HD duration, undergoing HDF, Kt/V_urea_, nPCR, non-anuria status, serum albumin levels, log iPTH levels, LDL levels, living in Taipei Basin, environmental NO_2_ levels, and environmental CO levels. To determine the association between residential areas and UP, multivariate forward logistic regression analyses of variables with a *P* of <0.1 in the univariate logistic regression (excluding environmental NO_2_ and CO levels) indicated that HD duration (OR, 1.1; 95% CI, 1.05–1.14; *P* < 0.001), non-anuria status (OR, 0.30; 95% CI, 0.15–0.59; *P* < 0.001), log ferritin levels (OR, 1.65; 95% CI, 1.02–2.69; *P* = 0.043), LDL levels (OR, 1.01; 95% CI, 1.01–1.02; *P* < 0.001), and living in Taipei Basin (OR, 32.93; 95% CI, 20.14–53.83; *P* < 0.001) were associated with UP (Table 3).

### Comparison of Clinical Variables between Patients Living in and around Taipei Basin

Patients living in Taipei Basin demonstrated a lower proportion of DM (13.1% vs 24.4%, respectively; *P* = 0.001) but demonstrated a higher proportion of UP (73.2% vs 9.5%, respectively; *P* < 0.001) and HDF use (27.4% vs 20.2%, respectively; *P* = 0.047) than patients living around Taipei Basin. Furthermore, patients living in Taipei Basin had a longer HD duration (8.71 ± 5.62 vs 6.54 ± 5.20 y, respectively; *P* < 0.001), higher Kt/V Daugirdes (1.88 ± 0.36 vs 1.77 ± 0.31, respectively; *P* < 0.001), higher nPCR (1.25 ± 0.27 vs 1.17 ± 0.26 g/kg/day, respectively; *P* < 0.001), higher iPTH levels (172.20 vs 123.40 pg/ml, respectively; *P* = 0.016), higher previous 12-month environmental NO_2_ levels (24.37 ± 6.47 vs 20.46 ± 5.57 ppb, respectively; *P* < 0.001), higher previous 12-month environmental CO levels (0.75 ± 0.38 vs 0.59 ± 0.29 ppm, respectively; *P* < 0.001), and higher previous 12-month environmental PM_2.5_ levels (29.60 ± 4.07 vs 28.32 ± 3.52 μg/m^3^, respectively; *P* < 0.001) than patients living around Taipei Basin (Table 4).

### Associations among UP, Clinical Variables, and Environmental Air Pollutants (NO_2_ and CO)

To further investigate the association between environmental air pollutants and UP, we used a multivariate logistic regression analysis to evaluate the association between mean previous 12-month and 24-month air pollutant levels (NO_2_ and CO) and UP in the patients. The result indicated that after adjusting for related factors (including BMI, HD durations, DM, HBV, HCV, HDF, Kt/V_urea_, nPCR, serum albumin levels, non-Anuria, corrected-calcium, Log iPTH, log ferritine, LDL levels, and mean previous 12- and 24-month Environmental PM_2.5_ level), UP in studied patients was positively associated with the mean previous 12-month environmental CO levels (OR, 1.73; 95% CI, 1.05–2.83; *P* = 0.03) and NO_2_ levels (OR, 1.05; 95% CI, 1.01–1.07; *P* = 0.001) levels (Table 5) and mean previous 24-month CO levels (OR, 1.82; 95% CI, 1.09–3.02; *P* = 0.02) and NO_2_ levels (OR, 1.05; 95% CI, 1.02–1.08; *P* = 0.001) (Table 6).

## Discussion

The present cross-sectional study indicates that environmental NO_2_ and CO levels are significant factors for UP in patients undergoing HD after adjusting for related factors. We also found that the residential environment is a factor associated with UP that is consistent with our previous study on the importance of living environment in dialysis patients^16^.

The mechanism of UP is not yet fully understood. A pro-inflammatory state secondary to immune system derangement with a high level of some cytokines, opioid-receptor system abnormalities, malnutrition, and dermal mast cells interfacing with the distal ends of unmyelinated C fibers, as well as possible mineral bone metabolism and nutritional disturbances, are among various mechanisms considered related to its causality or intensity^24^,^25^,^26^. Pruritus in MHD patients might occur possibly because skin barrier destruction induces an increase in neuron-specific enolase-immunoreactive nerve fibers in the epidermis^27^. It is well known that outdoor air pollution affects the health of patients with allergic diseases^28^,^29^,^30^,^31^. To our knowledge, this study is the first to show that environmental air pollutants (NO_2_ and CO) are positively associated with UP in MHD patients. Several studies have shown associations between air pollution and skin allergic disease through an inflammatory response^14^,^15^,^32^,^33^,^34^. The role of air pollutants on UP is unclear. It is likely that air pollutants aggravate skin symptoms such as pruritus, possibly by inducing oxidative stress in the skin that leads to skin barrier dysfunction and inducing an increase in neuron fibers in the epidermis, immune dysregulation, or neurogenic inflammation by environmental air pollutions^21^,^22^,^23^,^35^,^36^.

As for the studied air pollutants, NO_2_, an oxidant pollutant, induces oxidative damage to cell membranes, resulting in the generation of reactive oxygen species and subsequent inflammation, and could affect immunoglobulin levels, complement levels, and T-cell functions^37^,^38^,^39^,^40^. In a large cohort study, Morgenstern *et al*. demonstrated that the appearance of eczema in children is associated with environmental NO_2_ and other traffic-related air pollutant exposure levels^41^. Kim *et al*. also indicated that environmental NO_2_ concentrations and other air pollutant levels were associated with atopic dermatitis^14^.

Discussions on the association between environmental CO exposure and pruritus are rare. In a nationwide survery in 6- to 7-year-old children, Kim *et al*.^30^ presented that previous 12-month CO exposure levels were with high odds ratio for the presence of atopic dermatitis. The relationship between environmental CO and dermatits is not clear. However, environmental CO levels were associated with hsCRP levels in patients undergoing peritoneal dialysis^18^. In other studies, intermittent CO exposure caused damage to arterial walls and resulted in atherosclerosis^42^,^43^,^44^. A study by Davutoglu *et al*. demonstrated these cardiovascular effects and showed that hsCRP levels and carotid intima–media thickness were increased in subjects with chronic CO exposure^43^. Previously cited studies and our findings indicated that the role of environmental CO exposure in MHD patients may be explained by the relationship between inflammation and worsening pruritus^9^,^45^,^46^. In addition, in Kim’s study, high odds ratio (8.11) of atopic dermatitis was noted as 1 ppm increased CO level^30^. It is not clear why the difference of odds ratio between Kim’s study and ours. The reasonable explanation for above difference could be the different studied patients, underling diseases, age and living habits.

Interestingly, the odds ratio for patients with UP living in Taipei basin was about 32.93 (95% CI, 20.14–53.83); however, for environmental air pollutants (NO_2_ and CO), the odds ratio was around 1.8 for environmental CO levels and 1.05 for environmental NO_2_ levels in UP. In our study, the air pollutants levels for NO_2_, CO and PM_2.5_ in Taipei Basin were higher than those around Taipei Basin. To our knowledge, there are several environmental factors including season, temperature, humidity, indoor pollutants levels, foods, contact irritants, and air pollutants levels that are associated with allergic disease or allergic sensitization^14^,^15^,^21^,^32^,^34^. In our study, the environmental PM_2.5_ level was positively associated with UP, but without significance (*P* > 0.05). A reasonable explanation might be that in this study, we did not include all environmental allergic factors (unavailable) such as other pollutants, humidity, season and temperature, and it seems likely that other environmental factors that we did not include in this study might be correlated with UP. From above, we also could explain why the large gaps in the odds ratio between residential area and air pollutants (CO and NO_2_). Although this study was designed as a cross-sectional study, we used the average previous 12-month and 24-month air pollutant levels including CO, NO_2_, and PM_2.5_ for our analysis of UP that seems to be similar to a semi-cohort–designed study. Our study suggested that chronic exposure to environmental air pollutants (NO_2_ and CO) might be associated with pruritus in patients with MHD.

In this study, HD duration is a factor associated with UP that is consistent with several studies^47^,^48^. However, a study by Malekmakan did not demonstrate this result^45^. In our study, patients who lived in Taipei Basin had a longer HD duration than those who lived around Taipei Basin. From discussion of previous study^16^, comparison of living environment between city and suburb situations is complex. Air pollution is the one factor that could be quantified efficiently. Exactly, it is the matter of international concern. The Taipei basin is surrounded by hills and mountains, and about 6 million people living in it. So, the complexity of the terrain increases the difficulty of the diffusion of air pollution. From above, long-term contact with inflammation-inducing substances such as environmental air pollutants (NO_2_ and CO) could explain the prevalence of UP in patients living in different areas.

This study had some limitations. First, we did not include other air pollutants and other environmental factors such as temperature, humidity, seasons, diets, mite or contract irritants. It means that we could have under- or overestimated the correlation between environmental air pollutants (NO_2_ and CO) and UP in this study. We also know that UP is difficult to treat and study on pruritus of HD patients is complicated because of above mentioned factors. In the treatment of UP, in our dialysis centers, we also offer many methods to avoid pruritus that we know. Second, this was a cross-sectional study, and we only found the correlation between environmental CO/NO_2_ and UP, not the relationship between cause and effect. Third, air pollution data were collected and calculated from the previous 12 months and 24 months. In several studies on the association between air pollution and allergic disease, the duration of observation is 12-month^28^,^29^,^30^,^31^. In this study, use of previous 24-month air pollutants levels for analysis additionally is consistent with the effects of previous 12-month environmental air pollutants levels.

## Conclusion

This cross-sectional study showed that environmental air pollutants (NO_2_ and CO) might be a factor associated with UP in patients on MHD. Further, prevalence of UP may differ based on location. Further studies are required to clarify the role of other air pollutants or environmental factors on UP in patients undergoing MHD.

## Patients and Methods

### Methods

The Institutional Review Board Committee of Chang Gung Memorial Hospital approved the study protocol. Informed consent was obtained from all patients. All medical records during the study period, including medical history, laboratory data, and inclusion and exclusion factors, were reviewed by senior nephrologists. In addition, all individual information was securely protected and only available to the investigators. And all experiments protocols were conducted according to the Strengthening the Reporting of Observational Studies in Epidemiology guidelines.

### Patients

**Figure 1:**
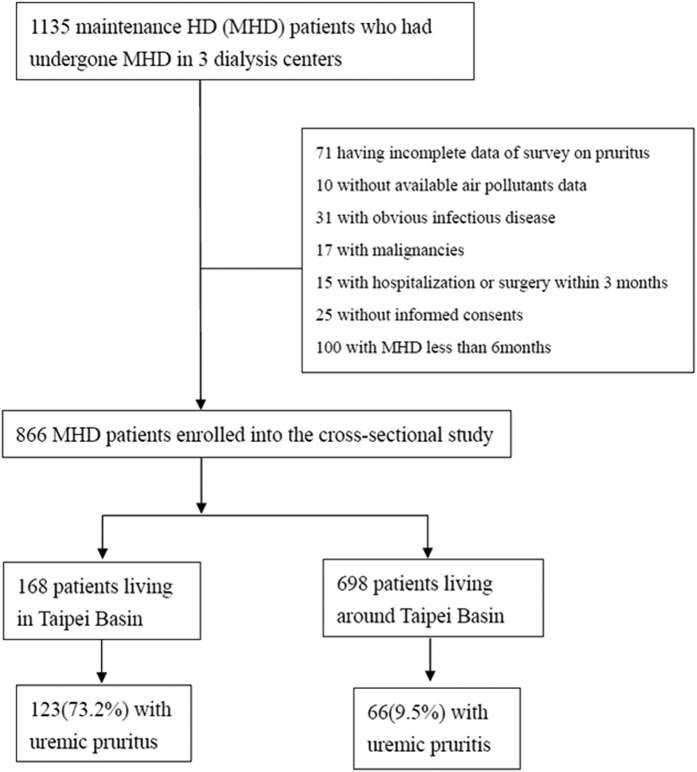
Flow chart shows enrollment and status of patients. MHD: maintenance hemodialysis.

This is a random-selection, cross-sectional study on patients undergoing MHD in 3 hemodialysis centers. Recruitment started in February 2013, and follow-up ended in December 2013. Study patients were recruited from the 3 hemodialysis centers of Chang Gung Memorial Hospital, Lin-Kou Medical Center at both the Taipei and Taoyuan branches. Only MHD patients who were 18 years of age or older and had undergone HD for at least 6 months were enrolled in this study. Residential areas were divided as follows: in Taipei Basin and around Taipei Basin. Coverage of Taipei Basin included: East, Nankang District of Taipei City; West, Shulin District of New Taipei City; South, Jingmei District of Taipei City; and North, Beitou District of Taipei City^16^. To be included in this study, patients had to be undergoing hemodialfiltration (HDF) 3 times weekly for ≥3 months. Patients with malignancies or obvious infectious diseases, as well as those who had been hospitalized or had undergone surgery within 3 months of the investigation, were excluded (Fig. 1). Diabetes mellitus (DM) was defined by either a physician’s diagnosis and antidiabetic drug treatment or 2 subsequent analyses demonstrated fasting blood glucose levels of >126 mg/dL. Most patients (98.2%) underwent 4 h of HD 3 times a week. HD was performed using single-use hollow-fiber dialyzers equipped with modified cellulose, polyamide, or polysulfone membranes. The dialysate used in all cases had a standard ionic composition with a bicarbonate-based buffer. We noted the incidence of cardiovascular diseases (CVDs) including cerebrovascular disease, coronary artery disease, congestive heart failure, and peripheral vascular disease in these patients. Hypertension was defined as the regular use of antihypertensive drugs to control blood pressure or at least 2 blood pressure measurements of more than 140/90 mmHg. Smoking behavior was also noted in this study. Diagnosis of pruritus was as follows: pruritus appearing after HD with/without antipruritics as visualized by trained dermatologists or nephrologists.

There are several air pollutants which are monitored for air quality control such as particulate matter with aerodynamic diameter less than 10 and 2.5 mm (PM_10_ and PM_2.5_), ozone (O_3_), sulfur dioxide (SO_2_), nitrogen dioxide (NO_2_) and carbon monoxide (CO). From our previous studies^17^,^18^,^19^,^20^, we found the association between environmental CO levels and inflammation in peritoneal dialysis (PD) patients, between environmental NO_2_ levels and mortality in PD patients, and between environmental particulate matter and PD-related infections in PD patients and arterial sclerosis in HD patients; therefore, we analyzed the association of above mentioned air pollutants (environmental NO_2_, CO, and PM_2.5_) levels in UP. Individual exposure to air pollution was estimated by a geographic information system using the mean previous 12- and 24-month concentrations of air pollutants.

### Laboratory, Nutritional, and Inflammatory Parameters

All blood samples were drawn from the arterial end of the vascular access immediately after the initial 2-day interval for HD and were then centrifuged and stored at −80 °C until use. Serum creatinine levels, normalized protein catabolism rate (nPCR), and serum albumin levels were assayed and recorded as nutritional markers. High-sensitivity C-reactive protein (hsCRP) levels were measured as indices of inflammation. The serum hsCRP concentration was measured using immunonephelometry (Nanopia CRP; Daiichi Inc, Tokyo, Japan). The lowest detection limit was <0.15 mg/L. All other biochemical indices were measured using a standard laboratory approach with an automatic analyzer. In HD patients, dialyzer clearance of urea was measured using a method described by Daugirdas and was expressed as Kt/V urea^49^. The nPCR of the HD patients was calculated using validated equations and was normalized to their body weight^50^. The serum calcium level was corrected using the serum albumin level with the following formula: corrected calcium level (mg/dL) = serum calcium level + 0.8 × (4.0 − serum albumin level). Non-anuria was defined as a daily urine amount of ≥100 mL.

### Statistical Analysis

The Kolmogorov–Smirnov test was used to test if variables were normally distributed. A *P* value of >0.05 was required to assume a normal distribution. Continuous variables are expressed as mean ± standard deviation/median (interquartile range), and categorical variables are expressed as numbers or percentages. *X*^2^ or Fisher’s exact test was used to analyze the correlation between categorical variables. Comparisons between 2 groups were performed using the Mann–Whitney *U* test or Student’s *t* test. Data involving hsCRP, intact parathyroid hormone, and ferritin levels were log-transformed for analysis. To evaluate the variables related to UP, univariate and multivariate (forward method) logistic regression analyses were performed to assess the odds ratio (OR) and 95% confidence interval (CI) for baseline variables including age, male sex, BMI, smoking status, DM, hypertension, previous CVDs, HBV infection, HCV infection, HD duration, blood access fistula, HDF, Kt/V_urea_ Daugirdes, nPCR, non-anuria status, hemoglobin levels, serum albumin levels, serum creatinine levels, corrected calcium levels, inorganic phosphate levels, log ferritin levels, log iPTH levels, log hsCRP levels, LDL levels, triglyceride levels, environmental NO_2_ levels (or environmental CO levels or environmental PM_2.5_ levels) (variables with a *P* value of <0.1 in the univariate logistic regression were selected for the multivariate logistic regression). According to the high collinearity (variance inflation factor [VIF]; NO_2_, 11.267; CO, 10.708) and high correlation between the environmental CO and NO_2_ levels, in the multivariate logistic regression, we included the 2 abovementioned items separately for the multivariate logistic regression analysis. All nominal variables in the logistic regression were transformed into dummy coding. Missing data were approached using list-wise deletion. Data were analyzed using SPSS, version 12.0 for Windows 95 (SPSS Inc, Chicago, IL). The level of significance was set at a *P* of <0.05.

## Additional Information

**How to cite this article**: Huang, W.-H. *et al*. Environmental NO_2_ and CO Exposure: Ignored Factors Associated with Uremic Pruritus in Patients Undergoing Hemodialysis. *Sci. Rep.* **6**, 31168; doi: 10.1038/srep31168 (2016).

## Figures and Tables

**Table 1:** Characteristics of studied MHD patients and with/without uremic pruritus.

Characteristics	Total (866) Mean ± SD/Median (IR)	Without pruritus (677) Mean ± SD/Median (IR)	With pruritus (189) Mean ± SD/Median (IR)	P
*Demographics*				
Age (years)	56.18 ± 13.59	55.70 ± 13.86	57.93 ± 12.43	0.046
Male sex (Yes)	440 (50.8%)	352 (52%)	88 (46.6%)	0.181
Body mass index (kg/m^2^)	22.19 ± 3.18	22.23 ± 3.13	22.06 ± 3.37	0.522
Smoking (Yes)	150 (17.3%)	120 (17.7%)	30 (15.9%)	0.581
Living in Taipei Basin	168 (19.4%)	45 (6.6%)	123 (65.1%)	<0.001
*Co-Morbidity*				
Diabetes mellitus (Yes)	192 (22.2%)	167 (24.7%)	25 (13.2%)	0.001
Hypertension (Yes)	339 (39.1%)	266 (39.3%)	73 (38.6%)	0.932
Previous CVD (Yes)	41 (4.7%)	34 (5%)	7 (3.7%)	0.561
HBV (Yes)	98 (11.3%)	84 (12.4%)	14 (7.4%)	0.063
HCV (Yes)	168 (19.4%)	121 (17.9%)	47 (24.9%)	0.037
*Dialysis Related Data*				
Haemodialysis duration (y)	6.96 ± 5.35	6.27 ± 5.13	9.41 ± 5.43	<0.001
Erythropoietin (U/kg/week)	73.62 ± 47.37	75.06 ± 47.71	68.45 ± 45.87	0.097
Fistula as blood access (Yes)	689 (79.6%)	532 (78.6%)	157 (83.1%)	0.182
Hemodiafiltration (Yes)	187 (21.6%)	135 (19.9%)	52 (27.5%)	0.028
Kt/V_urea_ Daugirdes	1.79 ± 0.32	1.77 ± 0.32	1.89 ± 0.34	<0.001
nPCR (g/kg/day)	1.18 ± 0.26	1.17 ± 0.26	1.22 ± 0.27	0.034
Residual daily urine of >100 ml	178 (20.6%)	156 (23%)	22 (11.6%)	<0.001
*Biochemical Data*				
Haemoglobin (g/dl)	10.51 ± 1.36	10.48 ± 1.34	10.62 ± 1.45	0.204
Albumin (g/dl)	4.06 ± 0.34	4.07 ± 0.34	4.01 ± 0.33	0.019
Creatinine (mg/dl)	10.88 ± 2.39	10.90 ± 2.42	10.84 ± 2.29	0.744
Ferritin (μg/l)*	305.0 (129.57, 504.45)	296 (116.60, 505.41)	335.2 (189.20, 499.40)	0.133
Corrected-calcium (mg/dl)	9.94 ± 0.93	9.91 ± 0.91	10.05 ± 0.97	0.055
Phosphate (mg/dl)	4.84 ± 1.35	4.84 ± 1.33	4.84 ± 1.41	0.991
Intact parathyroid hormone (pg/ml)*	130.1 (52.52, 319.2)	121.3 (47.7, 284.1)	187.2 (63.8, 401.7)	0.003
hsCRP (mg/l)*	2.95 (1.4, 7.01)	2.89 (1.38, 7.39)	3.04 (1.43, 5.94)	0.872
*Cardiovascular Risks*				
Cholesterol (mg/dl)	171.3 ± 37.66	169.73 ± 37.08	176.93 ± 39.24	0.021
Triglyceride (mg/dl)	164.33 ± 115.8	167.11 ± 118.67	154.36 ± 104.57	0.183
LDL (mg/dl)	94.83 ± 30.59	93.26 ± 30.30	100.36 ± 31.05	0.005

Abbreviations: IR: interquartile range. CVD: cardiovascular disease. HBV: hepatitis B virus infection. HCV: hepatitis C virus infection. nPCR, normalized protein catabolic rate. hsCRP = high-sensitivity C-reactive protein, LDL = low density lipoprotein. Kt/V _urea_ = dialysis clearance of urea.

^*^Non-normal distribution data are presented as median (interquartile range).

**Table 2:** Univariate logistic regression analysis between uremic pruritus and clinical variables.

Characteristics	Univariate logistic regression	p
*Variables*	Odds ratio (OR) 95% confidence Intervals (CI)	
Age (years)	1.01 (0.99–1.02)	0.188
Male sex	1.21 (0.71–2.09)	0.476
Body mass index (kg/m^2^)	1.07 (1.01–1.15)	0.036
Smoking (Yes)	1.32 (0.77–2.26)	0.300
Diabetes mellitus (Yes)	0.46 (0.29–0.73)	0.001
Hypertension (Yes)	1.16 (0.80–1.68)	0.422
Previous CVD (Yes)	0.86 (0.33–2.18)	0.752
HBV (Yes)	0.56 (0.31–1.01)	0.058
HCV (Yes)	1.52 (1.03–2.23)	0.032
Haemodialysis duration (years)	1.11 (1.05–1.15)	<0.001
Fistula as blood access (Yes)	1.21 (0.75–1.96)	0.428
Hemodiafiltration (Yes)	1.52 (1.05–2.20)	0.026
Kt/V_urea_ (Daugirdes)	2.94 (1.8–4.79)	<0.001
nPCR (g/kg/day)	1.93 (1.06–3.51)	0.03
Non-Anuria	0.44 (0.27–0.71)	0.001
Haemoglobin (g/dl)	1.12 (0.96–1.31)	0.160
Serum Albumin (g/dl)	0.57 (0.36–0.91)	0.02
Creatinine (mg/dl)	1.09 (0.96–1.24)	0.205
Corrected-calcium (mg/dl)	1.18 (0.99–1.40)	0.056
Phosphate (mg/dl)	0.99 (0.84–1.16)	0.860
Log ferritin	1.37 (0.96–1.95)	0.079
Log iPTH	1.52 (1.14–2.02)	0.004
Log hsCRP	0.78 (0.52–1.18)	0.231
Cholesterol (mg/dl)	1.01 (1.00–1.01)	0.021
Triglyceride (mg/dl)	0.99 (0.99–1.00)	0.215
LDL (mg/dl)	1.01 (1.00–1.01)	0.005
Living in Taipei Basin (yes)	26.17 (17.11–40.05)	<0.001
Mean previous 12-month Environmental NO_2_ (ppb)	1.05 (1.02–1.08)	<0.001
Mean previous 12-month Environmental CO (ppm)	1.87 (1.18–2.94)	0.007
Mean previous 12-month Environmental PM_2.5_ (ug/m^3^)	1.04 (0.99–1.09)	0.061

Abbreviations: MHD: maintenance haemodialysis. HBV: hepatitis B virus infection. HCV: hepatitis C virus infection. nPCR, normalized protein catabolic rate. iPTH: intact-parathyroid hormone. hsCRP = high-sensitivity C-reactive protein, LDL = low density lipoprotein. Kt/V _urea_ = dialysis clearance of urea.

**Table 3:** Multivariate logistic regression analysis (forward method) between uremic pruritus, and residential areas and clinical variables.

#*Variables*	Multivariate logistic regression	p
	Odds ratio (OR) 95% confidence Intervals (CI)	
Haemodialysis duration (years)	1.1 (1.05–1.14)	<0.001
Non-Anuria	0.30 (0.15–0.59)	<0.001
Log ferritin	1.65 (1.02–2.69)	0.043
LDL (mg/dl)	1.01 (1.01–1.02)	<0.001
Living in Taipei Basin (yes)	32.93 (20.14–53.83)	<0.001

^#^After adjustment for body mass index, DM, HBV, HCV, hemodiafiltration, Kt/V_urea_, nPCR, Serum Albumin, Corrected-calcium, and Log iPTH.

Abbreviations: MHD: maintenance haemodialysis. HBV: hepatitis B virus infection. HCV: hepatitis C virus infection. nPCR, normalized protein catabolic rate. iPTH: intact-parathyroid hormone. hsCRP = high-sensitivity C-reactive protein, LDL = low density lipoprotein. Kt/V _urea_ = dialysis clearance of urea.

**Table 4:** Comparison of patients living around Taipei Basin and living in Taipei Basin.

Characteristics	Around Taipei basin (698) Mean ± SD/Median (IR)	Taipei basin (168) Mean ± SD/Median (IR)	P
*Demographics*			
Age (y)	56.10 ± 13.65	56.52 ± 13.40	0.71
Male sex	355 (50.9%)	85 (50.6%)	0.99
Body mass index (kg/m^2^)	22.20 ± 3.18	22.15 ± 3.19	0.85
Smoking (Yes)	122 (17.5%)	28 (16.7%)	0.91
*Co-Morbidity*			
Diabetes mellitus (Yes)	170 (24.4%)	22 (13.1%)	0.001
Hypertension (Yes)	272 (39%)	67 (39.9%)	0.86
Previous CVD (Yes)	35 (5%)	6 (3.6%)	0.54
HBV (Yes)	83 (11.9%)	15 (8.9%)	0.34
HCV (Yes)	129 (18.5%)	39 (23.2%)	0.19
Pruritus (yes)	66 (9.5%)	123 (73.2%)	<0.001
*Dialysis Related Data*			
Haemodialysis duration (y)	6.54 ± 5.20	8.71 ± 5.62	<0.001
Fistula as blood access (Yes)	549 (78.7%)	140 (83.3%)	0.2
Hemodiafiltration (Yes)	141 (20.2%)	46 (27.4%)	0.047
Kt/V Daugirdes	1.77 ± 0.31	1.88 ± 0.36	<0.001
nPCR (g/kg/day)	1.17 ± 0.26	1.25 ± 0.27	<0.001
Residual daily urine of >100 ml	142 (20.3%)	36 (21.4%)	0.75
*Biochemical Data*			
Haemoglobin (g/dl)	10.47 ± 1.35	10.68 ± 1.40	0.076
Albumin (g/dl)	4.07 ± 0.34	4.03 ± 0.34	0.253
Creatinine (mg/dl)	10.89 ± 2.42	10.83 ± 2.25	0.75
Ferritin (μg/l)*	305.95 (117.1, 506.85)	301.95 (186.05, 489.87)	0.63
Corrected-calcium (mg/dl)	9.94 ± 0.93	9.92 ± 0.93	0.8
Phosphate (mg/dl)	4.86 ± 1.35	4.75 ± 1.35	0.34
iPTH (pg/ml)*	123.40 (48.92, 289.97)	172.20 (67, 425.82)	0.016
hsCRP (mg/l)*	3.01 (1.45, 7.51)	2.56 (1.27, 5.56)	0.14
*Cardiovascular Risks*			
Cholesterol (mg/dl)	171.17 ± 37.31	171.86 ± 39.21	0.83
Triglyceride (mg/dl)	164.22 ± 115.60	164.74 ± 116.99	0.95
LDL (mg/dl)	94.86 ± 30.49	94.72 ± 31.09	0.95
Previous 12-month Environmental NO_2_ (ppb)	20.46 ± 5.57	24.37 ± 6.47	<0.001
Previous 12-month Environmental CO (ppm)	0.59 ± 0.29	0.75 ± 0.38	<0.001
Previous 12-month Environmental PM_2.5_ (ug/m^3^)	28.32 ± 3.52	29.60 ± 4.07	<0.001

Abbreviations: IR: interquartile range. MHD: maintenance haemodialysis. HBV: hepatitis B virus infection. HCV: hepatitis C virus infection. nPCR, normalized protein catabolic rate. iPTH: intact-parathyroid hormone. hsCRP = high-sensitivity C-reactive protein, LDL = low density lipoprotein. Kt/V _urea_ = dialysis clearance of urea.

^*^Non-normal distribution data are presented as median (interquartile range).

**Table 5:** Multivariate logistic regression analysis (forward method) between uremic pruritus and mean previous 12-month environmental CO, NO_2_ level and other variables (p < 0.1 in univariae logistic regression were included).

#*Variables*	Multivariate logistic regression *(excluding NO_2_)	p	Multivariate logistic regression *(excluding CO)	p
	Odds ratio (OR) 95% confidence Intervals (CI)		Odds ratio (OR) 95% confidence Intervals (CI)	
Haemodialysis duration (years)	1.11 (1.08–1.15)	<0.001	1.11 (1.08–1.15)	<0.001
Serum Albumin (g/dl)	0.59 (0.35–0.97)	0.041	0.58 (0.35–0.97)	0.039
Log ferritin	1.77 (1.21–2.61)	0.003	1.78 (1.21–2.62)	0.003
LDL (mg/dl)	1.01 (1.00–1.01)	0.003	1.01 (1.00–1.01)	0.004
Mean previous 12-month Environmental NO_2_ (ppb)			1.05 (1.01–1.07)	0.001
Mean previous 12-month Environmental CO (ppm)	1.73 (1.05–2.83)	0.03		

^#^After adjustment for body mass index, DM, HBV, HCV, hemodiafiltration, Kt/V_urea_, nPCR, Non-Anuria, Corrected-calcium, Log iPTH, and mean previous 12-month Environmental PM_2.5_ level. *According to the high collinearity (variance inflation factor [VIF]; NO_2_: 11.267; CO: 10.708) and high correlation between environmental CO and NO_2_ levels, in the mode of multivariate logistic regression, we included NO_2_ and CO separately for multivariate logistic regression analysis.

Abbreviations: MHD: maintenance haemodialysis. HBV: hepatitis B virus infection. HCV: hepatitis C virus infection. nPCR, normalized protein catabolic rate. iPTH: intact-parathyroid hormone. hsCRP = high-sensitivity C-reactive protein, LDL = low density lipoprotein. Kt/V _urea_ = dialysis clearance of urea.

**Table 6:** Multivariate logistic regression analysis (forward method) between uremic pruritus and mean previous 24-month environmental CO, NO_2_ level and other variables (p < 0.1 in univariae logistic regression were included).

#*Variables*	Multivariate logistic regression *(excluding NO_2_)	p	Multivariate logistic regression *(excluding CO)	p
	Odds ratio (OR) 95% confidence Intervals (CI)		Odds ratio (OR) 95% confidence Intervals (CI)	
Haemodialysis duration (years)	1.11 (1.08–1.15)	<0.001	1.11 (1.08–1.15)	<0.001
Serum Albumin (g/dl)	0.59 (0.35–0.98)	0.041	0.59 (0.35–0.98)	0.042
Log ferritin	1.77 (1.21–2.61)	0.003	1.77 (1.20–2.61)	0.004
LDL (mg/dl)	1.01 (1.00–1.01)	0.003	1.01 (1.00–1.01)	0.004
Mean previous 24-month Environmental NO_2_ (ppb)			1.05 (1.02–1.08)	0.001
Mean previous 24-month Environmental CO (ppm)	1.82 (1.09–3.02)	0.020		

^#^After adjustment for body mass index, DM, HBV, HCV, hemodiafiltration, Kt/V_urea_, nPCR, Non-Anuria, Corrected-calcium, Log iPTH, and mean previous 24-month Environmental PM_2.5_ level.

^*^According to the high collinearity (variance inflation factor [VIF]; NO_2_: 11.267; CO: 10.708) and high correlation between environmental CO and NO_2_ levels, in the mode of multivariate logistic regression, we included NO_2_ and CO separately for multivariate logistic regression analysis.

Abbreviations: MHD: maintenance haemodialysis. HBV: hepatitis B virus infection. HCV: hepatitis C virus infection. nPCR, normalized protein catabolic rate. iPTH: intact-parathyroid hormone. hsCRP = high-sensitivity C-reactive protein, LDL = low density lipoprotein. Kt/V _urea_ = dialysis clearance of urea.
